# A Critical Review on the Relevance of Paracetamol for Procedural Pain Management in Neonates

**DOI:** 10.3389/fped.2020.00089

**Published:** 2020-03-18

**Authors:** Karel Allegaert

**Affiliations:** ^1^Development and Regeneration, KU Leuven, Leuven, Belgium; ^2^Pharmaceutical and Pharmacological Sciences, KU Leuven, Leuven, Belgium; ^3^Clinical Pharmacy, Erasmus MC Rotterdam, Rotterdam, Netherlands

**Keywords:** acetaminophen, paracetamol, newborn, infant, procedural pain management, opioid sparing, pain prevention, pain treatment

## Abstract

Effective and safe pain relief in neonates matters. This is not only because of ethical constraints or human empathy, but even more because pain treatment is an important and crucial part of contemporary medical, paramedical, and nursing care to improve the outcome in neonatal intensive care graduates. Paracetamol (acetaminophen) is likely one of the pharmacological tools to attain this, with data on prescription practices suggesting that paracetamol is somehow the “rising star” in neonatal pain management. Besides very rare topical clinical scenarios like peripartal asphyxia and subsequent whole body hypothermia or the use of cardiorespiratory support devices, data on paracetamol pharmacokinetics and metabolism were reported throughout neonatal age or weight ranges, and we have summarized these data. In this review, we subsequently aimed to provide the reader with the currently available observations on the use of paracetamol as analgesic for different pain syndromes (major surgery, minor surgery or trauma, and procedural pain), with focus on the limitations of paracetamol when prescribed for neonatal procedural pain management. We hereby intentionally will not discuss other indications (patent ductus arteriosus and fever) for paracetamol administration in neonates. Based on the available evidence, paracetamol has opioid-sparing effects for major pain syndromes, is effective to treat minor to moderate pain syndromes, but fails for effective procedural pain management in neonates. This efficacy failure for procedural pain management should stimulate us to continue to search for more effective interventions, including non-pharmacological interventions and preventive strategies. Furthermore, there are also upcoming association type of epidemiological studies on the relation between exposure to analgesics—including paracetamol—and the negative short- or long-term outcome characteristics (neuro-behavioral, atopy, and fertility). Consequently and in addition to the search for effective alternatives to prevent or treat pain, studies on long-term outcome following paracetamol exposure are needed to inform all stakeholders on the full effect–side effect balance of the different strategies to treat pain.

## Introduction

More than 30 years ago, the “credo” that immaturity protects (pre)term infants from pain sensations and its side effects got rejected by Anand et al. as he reported that untreated (i.e., surgery without opioids) pain during surgery (using the patent ductus arteriosus ligation in preterm neonates as a model) resulted in both increased mortality and morbidity (like endocrine stress response or post-operative infections). Moreover, these side effects were not only restricted to the neonatal admission as it also was more recently published that the impact of inadequate pain handling was also observed in later infancy and beyond (1, 2). Consequently, effective pain management in neonates matters. This is not only related to ethical constraints or human empathy, but even more because pain treatment is an important and crucial part of contemporary medical, paramedical, and nursing care to improve the outcome in neonatal intensive care graduates.

Inadequate management of pain in (pre)term neonates changes or affects the thresholds of pain, pain or stress-related behavior, and physiological responses and contributes to impaired neurodevelopmental outcome. The maturational development of the peripheral and central nervous system is driven by a mix of processes and mechanisms, including proliferation of cells, their subsequent migration and differentiation, and also selective cell death, with apoptosis as a related mechanism. These anatomic findings have their functional correlates in the physiological balance between the excitatory and the inhibitory interactions between cells. Because the maturational changes throughout infancy are associated with plasticity over time of these nociceptive systems, the excitatory input as a consequence of the nociceptive input may result in population-specific, lasting alterations in pain processing patterns (3–6). Based on the very same concept of a physiological dysbalance between the excitatory and the inhibitory interactions between neurons, it is reasonable to anticipate that (over)exposure to analgesics will likewise result in (over)inhibition of these interactions, especially in a setting of absence of excitatory-related pain. Although this is still mainly based on animal experimental observations, this can also result in poorer neurodevelopmental outcome in neonates (7).

In the human (pre)term newborn, there are arguments that the limbic system displays a given vulnerability for overexposure to pain, stress, or drugs, like analgesics or sedatives. This is likely because the maturational changes in the limbic structures evolve at a very fast rate throughout the last trimester of pregnancy until late infancy. It is well-known that the limbic system, with the hippocampus and the regions connected to the hippocampus, is essential as a functional switch board to encode, consolidate, and retrieve memory. Intriguingly, these type of memory deficits are frequently observed in former neonatal intensive care (NICU) graduates (8). These long-term neurodevelopmental outcome findings should be considered when decisions are made on the balance between efficacy and safety. At best, this should be combined with the published observations on the short-term side effects of narcotics—mainly morphine—that were reported as secondary outcome variables of the placebo-controlled trials on pre-emptive morphine administration in preterm neonates that undergo ventilation.

The reported short-term side effects associated to opioid exposure in preterm neonates include hypoventilation and apnea, low blood pressure, intestinal hypoperistalsis, and bladder dysfunction. Hypoventilation and apnea resulted in prolonged duration [7 (4–20) days in morphine-exposed group compared to 6 (3–19) days in the placebo group, + 1 day of ventilation] (9). Along the same line, Hartley et al. recently reported that morphine (single oral, 100 μg/kg dose) in non-ventilated preterm infants, used to facilitate the screening for retinopathy of prematurity (ROP) in the Procedural Pain in Premature Infants (POPPI study) and to blunt the pain response, resulted in a high incidence (8/15 vs. 3/15, relative risk 2.7, number needed to harm = 3) of either newly occurring apneic events or an increase in the number of such events in the morphine-exposed group (10). Because of these safety-related findings (sufficient evidence of harm), the POPPI study was terminated early (10). The available data on the severity and the incidence of low blood pressure in the different studies also likely reflect differences in morphine doses and subsequent morphine exposure. In the NEOPAIN study, the morphine maintenance (10–30 μg/kg/h) dose, the use of additional morphine boluses, and the immaturity (reflected by a younger age in cases) were associated with a higher risk to observe low blood pressure as an adverse event (11). In contrast, Simons et al. (morphine maintenance infusion, 10 μg/kg/h) did not observe lower blood pressure in morphine-exposed preterm neonates nor in inotropic prescription practices, irrespective of the post-menstrual age, within their randomized, placebo-controlled study (12). Finally, morphine delayed the attainment of full enteral nutrition (secondary analysis) by 3 days [17 (12–26) in the placebo arm compared to 20 (13–29) days in the morphine arm] in the NEOPAIN trial (13).

Consequently, a balanced approach, considering both wanted and unwanted effects, is appropriate, and data on drug utilization of analgesics hereby provide us some insights in the epidemiology and trends of drug prescription practices, and this also includes paracetamol (14–17).

## Epidemiology of Drug Prescription Practices of Analgesics in Neonates

Research on prescription practices provide us with information on *trends over time*, on *between unit variability*, or *heterogeneity in practices* and on *to what extent impact guideline development and implementation* changes these practices. At present, such data are available for opioid use in neonates but are still fragmented for paracetamol.

The Pediatrix group has a tradition to report on prescription practices and its trends within their network. When we compare two consecutive cohorts (1997–2004 vs. 2005–2010), fentanyl and morphine were in the top 30 list (19/30 and 25/30, prescriptions observed in 56/1,000 and 35/1,000 hospitalized neonates, respectively) in the first cohort (1997–2004) to rise to position 7 and 14 (exposure observed in 70 and 51/1,000 admitted neonates) in the more recently (2005–2010) treated cohort (14, 15). In another analysis of the same Pediatrix database with focus on 8,591 preterm (<32 weeks gestational age) ventilated neonates, opioid prescription rose from 5 to 32% of the days on ventilation over the time interval. This increase is observed despite the fact that there is a meta-analytical, Cochrane level of evidence not to prescribe opioids systematically in this setting, but rather “as needed” (17, 18). Besides the increase, the Canadian network and the EUROPAIN cohort also reported on the heterogeneity in prescription patterns between different units. In the Canadian network, an extensive between-unit variability in exposure (3 to 41% of admissions) to analgesics was observed, and these differences were not explained by clinical characteristics like surgery (19, 20). A similar pattern, including regular (26%) opioid prescriptions and significant between-unit variability in practices, was observed in the EUROPAIN study (243 units, 6,680 neonates) (19, 20).

Finally, the implementation efforts resulting in the bedside implementation of structured guidance on opioid prescription are effective to lower the use and the variability between units (21). This reduction in morphine prescription can be quantified by the number of exposed neonates (33% instead of 63%, −47%) or the cumulative morphine (0.51 mg/kg instead of 1.64 mg/kg, −68%) dose in opioid-exposed neonates. To further stress the clinical relevance of such implementation efforts, this exercise also resulted in an important and statistically significant decrease (from 10/205 to 3/250 cases) in neonates for whom methadone was prescribed to treat iatrogenic neonatal abstinence syndrome (21). Rana et al. have not described their data on paracetamol prescriptions, but the paper supplement at least suggests that these pain treatment guidelines resulted in more paracetamol prescription, both when considering the cases exposed as well as the duration of exposure in these cases (21).

As pain management in (pre)term neonates remains one of the core businesses of contemporary neonatal care and because of the side effects of opioids as mentioned earlier, there is hereby also a shift in practices to avoid opioids toward non-pharmacological interventions and paracetamol (22, 23). Data on prescription practices provide us with information but are still fragmented and anecdotic for paracetamol. The overall pattern hereby suggests that paracetamol is somehow a “rising star” in NICU pain management. This is reflected in the Pediatrix database mentioned earlier and the NEOPAIN study. While still absent in the top 30 in the first (1997–2004) cohort, paracetamol appeared in position 16 (43/1,000 prescriptions) in the more contemporary (2005–2010) cohort (15, 16). In the EUROPAIN study, paracetamol prescription (14%) was more common than that of sedatives/hypnotics (12%) but still lower when compared to opioid exposure (26%). Besides the guideline-driven decrease in morphine use as mentioned earlier and the increased paracetamol prescription of Rana et al., there is another recent paper on a clinical guideline-driven impact of reduced exposure to morphine and increased use of paracetamol (21, 24). Baarslag et al. reported that the adherence to a protocol that subsequently implemented intravenous paracetamol as a first-line treatment of pain after a major non-cardiac surgery in neonates and infants resulted in low (add-on) morphine needs, similar to the extent as published in the initial placebo-controlled randomized trial on the effect of intravenous paracetamol on post-operative morphine exposure (24, 25). In a recent report on post-surgery analgesic and sedative drug use in a single NICU (Lyon, France), paracetamol was routinely administered to 92% of the admitted neonates as part of a multi-modal analgesia approach (26).

Likely because of the uncertainties related to the pain assessment tools during the registration studies, intravenous paracetamol product registration for neonates failed in the United States, while labeling in Europe is limited to term neonates and beyond, not covering the needs of preterm neonates. Despite this setting, there is an important off-label prescription in (pre)term neonates (27, 28). In this review, we aim to provide the reader with the currently available data on the use of paracetamol as analgesic for the different pain syndromes (major surgery, minor surgery or trauma, and procedural pain), with focus on the limitations of paracetamol when prescribed for neonatal procedural pain management. We will hereby explicitly not discuss other indications (patent ductus arteriosus and fever). To put these findings into perspective, this is preceded by an overview on the pharmacology of paracetamol in this population and will be followed by a reflection on some more recently reported issues related to the safety of paracetamol with the intention to describe the currently available risk/benefit profile of this compound in the NICU setting.

## The Clinical Pharmacology of Paracetamol in Neonates

### Pharmacology and Clinical Pharmacology of Paracetamol

Paracetamol has pain- and fever-reducing effects, be it with only very modest anti-inflammatory properties in the peripheral tissues. Because of these modest peripheral properties, the effect compartment is better reflected by the cerebrospinal fluid compartment and not so much by the plasma compartment. The routes of administration are variable since this can be done by oral, rectal (enteral), or intravenous route.

Interestingly, after being marketed for almost 120 years, the mechanisms of actions for paracetamol are still only in part captured. There is concentration-dependent inhibition of the prostaglandin H2 synthetase (PGHS) enzyme. This PGHS complex has two sites: the cyclo-oxygenase (COX) site and the peroxidase (POX) site (28–31). Paracetamol hereby interacts with the POX enzyme as a reducing co-substrate so that less prostaglandin G_2_ will be altered to prostaglandin H_2_ at the POX site of this PGHS enzyme. Paracetamol-related POX co-substrate inhibition is competitive since it is in balance with prostaglandin G_2_ itself and by hydro-peroxides. This explains why the inhibition of prostaglandin synthesis is potent within the central nervous system (no lipid hydro-peroxides since the main sources of these peroxides are leukocytes and platelets). Paracetamol obviously has also the same competitive inhibition on peripheral COXs. However, this inhibitory action in peripheral tissues only occurs at physiological, normal low arachidonic acid concentrations as it is a competitive inhibition phenomenon. This also explains the difference between paracetamol and “real” non-steroidal anti-inflammatory drugs (NSAIDs) like ibuprofen or indomethacin. As their interaction with the COX enzyme is not competitive, NSAIDs has more potent anti-inflammatory peripheral effects, even more in an inflammatory (high hydro-peroxides and high prostaglandins) setting (30). The other mechanisms relate to the formation of an active metabolite (p-aminophenol) that interacts with cannabinoid receptors. The other mechanisms of paracetamol's pain-relieving effects are further mediated by descending serotonergic pathways activation, substance P-mediated processes, or interaction with the N-methyl D-aspartate (NMDA) receptor or nitrous oxide-related effects when acting as a spinal neurotransmitter (28–31).

In both adults and children, the paracetamol concentrations of 5 and 10 mg/L have been postulated for either temperature or pain relief (29). Although such target concentrations may be different in (pre)term neonates because of maturational differences, these targets are used to develop age-adapted and route-tailored dosing regimens. In this target concentration range, paracetamol is mainly metabolized by the liver into paracetamol-glucuronide (47–62%) and paracetamol-sulfate (25–36%) with subsequent renal excretion in adults and children. Only 1–4% is excreted unaltered as paracetamol in urine, while a minor route (8–10% of paracetamol) undergoes oxidation to 3-hydroxy-paracetamol to result in the toxic (liver and kidney) metabolite N-acetyl-p-benzoquinone-imine (NAPQI). Age- or weight-driven alterations in paracetamol pharmacokinetics and metabolism have been observed throughout pediatric life but are most relevant throughout infancy (first year of life). As a consequence, not only total clearance but also the different routes of elimination will change over age or weight. These changes necessitate the integration of this knowledge on age- or weight-specific paracetamol disposition data to targeted dosing and subsequent exposure before we can consider studying age category-specific pharmacodynamics (PD) (effects, but also for side effects). Issues on short-term safety and tolerance on paracetamol mainly relate to *hepatotoxicity* or *hemodynamics*, while long-term safety relate to neuro-behavioral (attention deficit hyperactivity disorder, autism spectrum disorders, and intelligence) outcome, atopy, or fertility (7, 27). Both aspects will be considered in the discussion part of the paper.

### Clinical Pharmacology of Paracetamol in Neonates

Clinical pharmacology wants to describe and predict the (side)effects based on the pharmacokinetics (PK) and pharmacodynamics for a given drug. PK (*a*bsorption, *d*istribution, *m*etabolism, and *e*xcretion, *ADME*) describes the concentration over time pattern (“what the body does to the drug”) in a given compartment, like plasma, subcutaneous tissue, or cerebrospinal fluid, for a given drug. PD describes the relationship between drug concentrations and (side)effects over time (“what the drug does to the body”) (32). For paracetamol, this can be illustrated by concentration–time profiles in plasma or cerebrospinal fluid for PK, while PD covers both effects (analgesia and fever) and side effects (blood pressure, hypothermia, and hepatic or renal impairment) (27, 33). Besides the mean or average values, clinical pharmacology also aims to describe the extent of variability and its covariates. This is where neonatal clinical pharmacology has its specific characteristics since differences between and within patients are the key and core characteristics of neonatal clinical pharmacology (34). This is also true for paracetamol PK and metabolism in (pre)term neonates.

Besides very rare but specific clinical scenarios like peripartal asphyxia and subsequent whole body hypothermia or the use of cardiorespiratory support devices, data on paracetamol pharmacokinetics and metabolism were reported throughout neonatal age or weight ranges. As recently suggested, physiologically based pharmacokinetic approaches should hereby be considered to facilitate and guide the clinical studies in these specific populations, incorporating both maturational and non-maturational covariates of variability in paracetamol disposition (35).

Based on the data on paracetamol pharmacokinetics and metabolism mentioned earlier and when aiming for the same target concentration as in adults or children (10 mg/L), we have suggested to use a loading dose (20 mg/kg, once) approach, to be followed by 10 mg/kg (q6h) of intravenous paracetamol in late (pre)term neonates (36). Aiming for the same target concentration, oral doses are similar (median estimates, not reflecting the additional absorption-related variability), with rectal administration of 25 to 30 mg/kg/day in preterm neonates of 30 weeks gestation, 45 mg/kg/day in preterm infants of 34 weeks gestation, and 60 mg/kg/day in term neonates suggested (all doses as suggested are off-label). Compared to the intravenous route, the subsequent peak concentration after oral dosing is observed about 1 h after administration. Absorption after rectal administration is even more variable, blunted, and delayed. The route-dependent impact can be explained by the route-related difference in bio-availability, while the suggestion to use a loading dose can be explained by the maturational changes in distribution volume (higher in more immature patients). Finally, the maturational changes in clearance are reflected in the age-dependent differences in maintenance doses.

#### Absorption

Pharmacokinetic datasets after enteral (rectal and oral) or intravenous administration were pooled to estimate the absorption patterns. Intravenous administration hereby bypasses the absorption-related variability associated with the oral or the rectal route (36–38). The bioavailability following rectal administration not only displayed age-dependent changes but also depended on the formulation used as—relative to elixir—the bioavailability of the solution was 0.66 (38). A decrease in bioavailability with increasing age from 0.92 (22%) at 28 weeks to 0.86 at 2 years was observed, whereas the triglyceride base formulation bioavailability decreased from 0.86 (35%) at 28 weeks to 0.5 at 2 years. Studies have repeatedly documented that the rectal route results in not only lower but also more erratic absorption compared to the oral route. Consequently, higher doses are needed when compared to oral administration, although it remains difficult to adapt dosing for the extent between observation and between individual variability (39, 40). As recently illustrated by Kleiber et al., the bioavailability after oral administration also displays variability (72, 11–91%) in young infants during intensive care admission (41).

#### Distribution

The relative distribution volume (L/kg) has a progressive decrease over time from 27 weeks post-menstrual age onwards to reach an adult-equivalent value for 6 months of age onwards (42, 43). As a consequence, the effect of a loading dose is over even more clinical relevance in early neonatal life. As their relative distribution volume (L/kg) for paracetamol is higher, the same mg/kg dose will result in a lower peak concentration in the setting of a higher distribution volume, while a lower peak concentration is likely associated with poorer pain control (43).

#### Metabolism and Elimination

Pharmacokinetic datasets were pooled to report on clearance and metabolism following either enteral (rectal and oral) or intravenous administration (36, 37, 42). Patient size (reflected in weight) is the dominant covariate to explain in-between variability in paracetamol clearance in early life. Using these pooled datasets, a mean target paracetamol of 11 mg/L in serum is anticipated in neonates (between 32 and 44 weeks post-menstrual age) when exposed to (maintenance doses and loading, *cf*. higher) intravenous paracetamol of 10 mg/kg (q6h) (36). Besides estimates on overall clearance, data on the different metabolic elimination routes (glucuronidation, sulfation, oxidation, and renal) were described and even prospectively confirmed (44–46). More recently, Flint et al. reported on the gestational age-determined raise in glucuronidation capacity in extreme preterm neonates. Importantly, this was without any evidence for saturation of a specific pathway like oxidation or sulfation (47).

## Paracetamol as Analgesic in Neonates: a Summary of the Available Evidence

A structured literature search was conducted in PubMed (November 2019, paracetamol + pain + newborn, preterm, or infant as keywords). This search resulted in 205, 33, and 468 hits, respectively. Following title, abstract, and full paper reading when perceived to be relevant to this review (this includes verification of references and a search for resulting citations), a summary of the available observations on the relevance of paracetamol as analgesic in neonates was constructed.

### Paracetamol and Opioid-Sparing Effects in Major Pain Syndromes in Neonates

There is meta-analytical evidence on the opioid-sparing effects of paracetamol administration to treat post-operative or post-traumatic pain in adults and in children. In contrast, the demonstration of a clinically significant reduction in opioid-related adverse effects (like sedation and nausea) is much less robust (48, 49). In contrast, data in neonatal cohorts on paracetamol-related opioid-sparing effects in neonates were only more recently reported in two different cohorts (surgical and medical intensive care, respectively). After a major non-cardiac surgery in (pre)term neonates, Ceelie et al. documented a clinically significant (−66%) morphine (maintenance dose) sparing effect in neonates and in infants co-exposed to intravenous paracetamol (25). In contrast, rectal paracetamol had no effect on morphine use after a major non-cardiac surgery in young infants, likely because of the variability in absorption and the doses used (90–100 mg/kg/day) (50). This assumption is further supported by a pilot study conducted in the same unit comparing intravenous (*n* = 12) vs. rectal (*n* = 14) paracetamol (both at 40 mg/kg/day) in young infants following a major craniofacial surgery. Intravenous paracetamol use proved to be more effective than rectal paracetamol using COMFORT-B scores (above 17, 9/14 vs. 3/14) as outcome variable of interest (51) (Table 1) (25, 50, 52). As discussed earlier, Baarslag et al. reported that protocol adherence to implement intravenous paracetamol as a first-line treatment of pain after a major non-cardiac surgery in neonates and infants resulted in low (add-on) morphine needs, similar to the extent as published in this placebo-controlled randomized trial.

**Table 1:** Studies on the relevance of paracetamol on pain (opioid reduction) during major pain syndromes in neonates (25, 50, 52).

**Reference**	**Study design, pain model**	**Paracetamol dosing**	**Results**
Van der Marel et al. (50)	Double-blind RCT, morphine + paracetamol or placebo, 30/54 PMA <45 weeks. Visual analog Scale (VAS) and comfort score. Major thoracic (lung, esophageal) or abdominal surgery	Rectal paracetamol 30–40 mg/kg loading dose, 20–30 mg/kg, q6h or q8h, maintenance dose	Cases < PMA 45 weeks needed less add on morphine compared to infants, but without any difference between both study arms (paracetamol or placebo) (threshold for additional morphine, ≥VAS)
Ceelie et al. (25)	Double-blind RCT, 71 neonates and infants (35/71 <10 days post-natal). Major thoracic (lung, esophageal) or abdominal surgery. Nurse rating scale-11 + comfort-B, cumulative maintenance dose of morphine was assessed	Morphine loading dose, followed by either continuous morphine or paracetamol i.v. (48 h). Backup morphine	No differences in pain scores Clinical relevant lower (−66%) exposure to morphine for the continuous morphine when compared to the paracetamol group
Härmä et al. (52)	Retrospective, “unblended” analysis (two consecutive time intervals) on opioid prescription in 218 preterm neonates (<32 weeks) before or following the use of intravenous paracetamol in the clinical pain management protocol (Neonatal Infant Acute Pain Assessment Scale) of a single Finnish unit	20 mg/kg intravenous as loading dose, followed by 7.5 mg/kg, q6h (maintenance)	Paracetamol-exposed neonates needed significantly fewer morphine doses, 1.78 [(4.56) vs. 4.35 (11.53)] and had a lower total morphine exposure [0.17 (0.45) vs. 0.37 (0.96) mg/kg]. No differences in pain score, days on ventilation of incidence of apneas

*RCT, randomized, controlled trial; PMA, post-menstrual age; LNPS, Leuven Neonatal Pain Scale; VAS, Visual Analog Scale*.

Along the same line but in a medical NICU setting, a reduction in opioid administration (−54% for cumulative dose and −59% for number of additional boluses) to extreme preterm neonates (<32 weeks) was documented following the systematic use of intravenous paracetamol as a first-line analgesic in a clinical pain management protocol of a single NICU (Oulu, Finland) (52).

### Paracetamol and Minor to Moderate Pain Syndromes in Neonates

Data on the impact of paracetamol to take care of minor to moderate pain syndromes in (pre)term neonates were quantified and reported following a “minor” surgery (one study, circumcision of 44 neonates) or following a birth-associated tissue damage [assisted vaginal delivery, bruising, three studies, 264 (pre)term neonates] in a randomized, placebo-controlled setting. In addition, there is also a retrospective analysis on the impact of intravenous paracetamol (prophylactic, for patent ductus arteriosus closure) on glucose 33% consumption and additional analgesics prescribed compared to a historical cohort (449 neonates) in a single (Innsbruck, Austria) NICU (Table 2) (53–57).

**Table 2:** Studies on the relevance of paracetamol on pain during minor to moderate pain syndromes in neonates (53–57).

**Reference**	**Study design, pain model**	**Paracetamol dosing**	**Results**
Van Lingen et al. (53)	RCT, 122 term neonates following instrumental vaginal delivery Facies pain scale + “gestalt” assessment	Rectal (20 mg/kg, q6h) for 24 h, either paracetamol or placebo after delivery	Similar Facies pain scores, objective clinical symptoms (nurse assessment) similar, except for the paracetamol group 1 h after first administration (better “gestalt” assessment)
Tinner et al. (54)	RCT, EDIN (Echelle de douleur et d'inconfort du nouveau né) score in the 24 h following instrumental vaginal delivery in 123 (near) term neonates. BPSN (Bernese pain score) following heel lancing on day 2–3 in the same neonates, with 0.2 ml sucrose in all cases.	Rectal paracetamol (20–25 mg/kg) 2 and 8 h after delivery	No differences in mean EDIN score, no differences in EDIN score ≥5. BPSN after heel prick higher in the “former” paracetamol group [5 (3–9) vs. 3 (0–6)]
Howard et al. (55)	RCT in 44 healthy term neonates that underwent neonatal circumcision (Gomco). Post-operative comfort score	Oral paracetamol 15 mg/kg, q6h for 24 h start 2 h before surgery	No effects during and immediately following circumcision. Post-operative score similar until 6 h, but paracetamol group scored better afterwards (>6 h).
Allegaert et al. (56)	Open-label intravenous paracetamol (19/60 mono-therapy). Instrumental vaginal delivery or bruising with Leuven Neonatal Pain Score (LNPS) indicating pain	Loading dose intravenous Paracetamol (20 mg/kg)	Decrease in LNPS from 0.5 h onwards, with a trend to return to these baseline scores at 5 h
Höck et al. (57)	Retrospective study design. Prophylactic paracetamol use in preterm (≤32 weeks, *n* = 221 vs. 228), in the first days of life, prophylactic to induce patent ductus arteriosus closure vs. historical data. Clinical pain assessment based on BNPS	Intravenous paracetamol (10 mg/kg, q8h)	Less 33% glucose use (protocol = to treat mild to moderate pain, mean 13.48 vs. 8.71 doses) in exposed cases, but no impact on the prescription of systemic analgesics

*RCT, randomized controlled trial; EDIN, Echélle de douleur en d'inconfort du nouveau-né; BPSN, Bernese pain score*.

Paracetamol (15 mg/kg, every 6 h for 24 h, oral) was ineffective as analgesic at and immediately after circumcision. However, there was some advantage (as the post-operative comfort score was lower compared to that of the controls) for the paracetamol-exposed cases afterwards (>6–24 h after the surgical intervention) (55). In a randomized, placebo-controlled study following assisted delivery (vacuum extraction and pre-emptive) in 122 term neonates, paracetamol (20 mg/kg, rectal, once) improved the initial clinical gestalt (e.g., drinking behavior), be it without any differences in the quantification of the pain as observed. Subsequent paracetamol dosing did not result in any difference in outcome, but neither was there any difference in side effect (53). In a very analog study in 123 (near)term neonates (20–25 mg/kg rectal route, 2 and 8 h post-natal age, pre-emptive), the pain scores remained low irrespective of paracetamol exposure following assisted vaginal delivery. However, the neonates that were initially exposed to paracetamol at delivery had a more vigorous and pronounced response (Bernese pain score) during heel lancing on post-natal days 2 and 3 (54). As mentioned, both studies used a pre-emptive prescription approach, so the initiated treatment was irrespective of the presence of pain at initiation. In contrast, an open-label study in neonates (*n* = 19) with pain (as assessed by a pain score) following a birth-related tissue damage resulted in lower pain scores within 0.5 h after the administration of intravenous paracetamol (20 mg/kg, loading dose), with a slight return toward these higher baseline scores after 5 h (56).

In our assessment, the available body of evidence suggest that paracetamol is likely effective in the presence of minor to moderate pain, but not when prescribed as part of a pre-emptive strategy. However, using a retrospective analysis, prophylactic “low”-dose paracetamol (10 mg/kg, q8h) administration in preterm (≤32 weeks) neonates with the intention to induce the closure of the patent ductus arteriosus was associated with a reduced prescription of 33% glucose (protocol = to treat mild to moderate pain) in exposed (mean 13.48 vs. 8.71 doses) cases, but without significant differences in the prescription of systemic analgesics (57).

### Paracetamol for Acute Procedural Analgesia in Neonates

It is important to realize that the available information strongly suggests that paracetamol fails to reduce acute procedural [skin-breaking procedures like heel lancing or peripherally inserted central catheter (PICC) placement and ROP screening] pain (58, 59). To further illustrate this, we have summarized the available information on heel lancing (60–63) and ROP (64–66) screening (Tables 3, 4).

**Table 3:** Studies on the relevance of paracetamol on pain during heel lancing in neonates (60–63).

**Reference**	**Study design, heel lancing**	**Paracetamol dosing**	**Results**
Shah et al. (60)	RCT, 75 full-term cases. Facial action pain scores and cry score.	Oral paracetamol, 20 mg/kg or placebo, once 1–1.5 h before prick	Similar facial action pain scores between both groups. Neither any difference in cry score
Bonetto et al. (61)	RCT, 76 full-term cases. Pain scores (NIPS, neonatal infant pain score >4)	Placebo, glucose (25%) EMLA or oral paracetamol (20 mg/kg, 60 min)	NIPS <4 similar between placebo, paracetamol, or EMLA (47, 42, and 63%). Oral dextrose most effective (84% NIPS <4, NNT 2.7)
Badiee et al. (62)	RCT, 72 preterm (mean 32 weeks) cases. PIPP (premature infant pain profile) score	High dose, oral paracetamol (40 mg/kg), once 1.5 h 90 before heel lancing	PIPP scores placebo were similar between placebo and paracetamol (9.7, SD 4.2 vs. 11.1, SD 3.8)
Foronda et al. (63)	Randomized, single-blinded study in 129 cases, NIPS, and PIPP score, paracetamol (42) to glucose (47) or placebo (40)	No data on the paracetamol dose provided	PIPP and NIPS score were lower, and crying time shorter in the glucose group No effect of paracetamol

*RCT, randomized controlled trial; ELMA, eutectic mixture of local anesthetics; NNT, number needed to treat; SD, standard deviation; NIPS, neonatal infant pain score; PIPP, premature infant pain profile*.

**Table 4:** Studies on the relevance of paracetamol on pain during retinopathy of prematurity (ROP) screening in neonates (64–66).

**Reference**	**Study design, ROP screening**	**Paracetamol dosing**	**Results**
Kabatas et al. (64)	RCT in 114 preterm neonates, screening retinopathy of prematurity. PIPP (premature infant pain profile) score	Topical anesthetics + paracetamol (15 mg/kg, oral, 60 min) or placebo	Significant lower PIPP in paracetamol exposed cases [12 (9–13) vs. 14 (13–15)], but very modest effect (cutoff PIPP score <7)
Seifi et al. (65)	RCT in 120 preterm neonates, screening retinopathy of prematurity. PIPP score	Sucrose 25% vs. oral paracetamol (15 mg/kg) vs. placebo	Sucrose was effective, paracetamol not when compared to placebo at eye examination
Manjunatha et al. (66)	RCT, examination retinopathy of prematurity 18 preterms recruited (63 intended), PIPP	Oral morphine (200 μg/kg) or paracetamol (20 mg/kg) or placebo	Morphine cases tended to have lower pain scores (PIPP), but no significant differences (underpowered study)

Table 3 (60–63) gives a condensed summary of the retrieved randomized studies (cf. search strategy described earlier) on paracetamol administration for procedural-related pain relief during heel lancing (352 cases, four studies). There was no benefit (improved pain control) in cases exposed to paracetamol when compared to placebo. When compared to non-pharmacological interventions (sucrose and dextrose), paracetamol-related analgesia was less effective to these non-pharmacological interventions. Similarly, Roofthooft et al. also concluded that intravenous paracetamol (10, 15, or 20 mg/kg) was not effective (PIPP score and Comfort-Neo) as an analgesic during PICC placement in 60 preterm (<32 weeks) neonates, irrespective of the dose administered (67). This is in line with similar findings on the absence of an analgesic effect of even high doses of paracetamol (40 mg/kg oral) on pain, fear, or distress as reported in a cohort of children with oncological diseases when they had to undergo a needle insertion into their subcutaneous implanted venous access (68). This suggests that, when considering the pain syndromes and the efficacy of paracetamol, neonates do not behave differently from children.

Table 4 illustrates the same limitations of paracetamol when assessed for its analgesic properties during an eye examination to screen for ROP (252 cases, three studies) in (pre)term neonates. Only one of these studies described a very modest decrease in median PIPP value (12 vs. 14), but not within the range usually aimed for to qualify an intervention as effective (64). Sucrose was effective compared to either paracetamol or placebo (65), and oral morphine (200 μg/kg) tended to result in lower pain scores. However, as mentioned earlier, in the POPPI study, oral morphine (100 μg/kg) was associated with either a higher incidence of newly occurring apneic events or an increase in the number of such events in the morphine-exposed group. Because of these safety-related findings (sufficient evidence of harm), the POPPI study was terminated early (10). Based on the currently available evidence, it seems that a non-pharmacological intervention, based on a bundled developmental care intervention concept, resulted in a clinically relevant reduction in the pain and stress responses and in the time needed to recover (69). It is therefore suggested that “combined” practices are likely the approach needed to make further progress or should at least be the background setting to assess the impact of additional interventions.

Paracetamol fails to reduce acute procedural pain in neonates (58, 59), but this should be put in some perspective. The observation on, e.g., circumcision instructs us that paracetamol does not improve pain scores in the first 6 h after the intervention, but the paracetamol group scored better afterwards (>6 h) (55). A similar pattern has been described for paracetamol use during and after immunization in infants. There is a significant benefit from paracetamol prophylaxis for fever and fussiness in the first 24 h after immunization, but not yet for analgesia during the needle-related procedure (70).

## Paracetamol Use in Neonates: in Search of a Better Benefit–Risk Balance

Pain management in (pre)term neonates remains one of the core businesses of contemporary neonatal care. Because of the early and delayed negative effects of opioids, there is a more recent shift in practices to avoid opioids and gear toward paracetamol or non-pharmacological interventions (22, 23). Related to this shift in practices, the available observations on paracetamol disposition and the dynamics of paracetamol in (pre)term neonates have “exploded” over the last two decades. We therefore summarized the evidence on the impact of paracetamol to treat major pain syndromes (effective to reduce opioid consumption) in minor to moderate pain syndromes (effective as monotherapy) or in procedural pain management (fails) in neonates. In search of a better benefit–risk balance, these findings should be further considered.

Firstly, the practice to administer paracetamol in settings where the available data strongly suggest that such practices are not effective should not result in established “common” practice (*efficacy*) and acceptance of a substandard setting but should stimulate us to further search for more effective and safer interventions. Secondly, avoidance or reduction of opioid exposure is a valid target, but it is perhaps too simple to assume that paracetamol is a benign drug without any side effects as it has effects on cytokine production that are only partly understood (*safety*) in the developmental context of the (pre)term neonate. Some epidemiological data and experimental studies in juvenile animals call for additional safety assessment studies.

Related to the needed search for more effective and safer interventions, this should, for sure, also include non-pharmacological interventions, like a care bundle to blunt the pain response during ROP screening (69). Even more relevant are technical adaptations in our practices and interventions that can be very effective to avoid or reduce pain. A venipuncture is more effective, is faster, and is associated with a less robust pain response compared to heel lancing (71); the use of a lens instead of an eye lid distractor results in a reduced pain response during ROP screening (72), while an assisted delivery with Kiwi OmniCup vs. metal ventouse is associated with a decreased neonatal pain response irrespective of the use of paracetamol (73).

Observations on short-term safety in (pre)term neonates related to hepatic injury (74), hemodynamic tolerance (75), and temperature regulation (76) have been reported (27, 74–76). Similar to other populations, N-acetyl-cysteine serves as a glutathione precursor to treat hepatotoxicity by reducing NAPQI production, and experience on its use in neonates has been summarized (27).

Long-term safety concerns relate to neuro-behavioral (attention deficit hyperactivity disorder, autism spectrum disorders, and intelligence) outcome, atopy, or fertility and are mainly based on epidemiological data analysis following maternal intake and subsequent fetal exposure. The neuro-behavioral epidemiological data are supported by postulated mechanisms and experimental observations in animals. Suggested mechanisms relate to modulation of central nervous system inflammation or to paracetamol metabolites, like cannabinoids. Related to this, paracetamol and Δ(9)-tetrahydrocannabinol, but not ibuprofen, resulted in developmental neurotoxicity in a mice model (77, 78). Paracetamol exposure also results in reduced COX-2 activity in the brain, as this is the crucial pharmacological effect for at least fever reduction and likely also for analgesia. These differences in phenotypic cerebral COX-2 activity are not only driven by drug exposure (paracetamol, ibuprofen, and indomethacin) but are also determined by genetic polymorphisms. Interestingly, the cognitive outcome in former preterm neonates is in part determined by COX-2 polymorphisms, with a lower polymorphism-determined phenotypic activity resulting in poorer outcome (79). The claimed mechanism related to atopy is COX-dependent, the inhibition of mucosal PGE2 synthesis, resulting in alteration of the maturational immunity (30). Finally, an association between maternal paracetamol intake and cryptorchidism or hypospadias has been described, with endocrine disturbances as the mechanism (80).

Both the Food and Drug Administration and the European Medicine Agency assessed the reported datasets in 2015 and in 2019, respectively, and concluded that the clinical translation of these potential associations remains uncertain. This leads to the decision not to adapt their guidance, while the leaflets (summary of product characteristics, SmPC) were changed in the specific section on fertility, lactation, and pregnancy (verbatim copied from SmPC, adapted wording is underlined and in italics: “A large amount of data on pregnant women indicate neither malformative, nor feto/neonatal toxicity. *Epidemiological studies on neurodevelopment in children exposed to paracetamol in utero show inconclusive results. If clinically needed, paracetamol* can be used during pregnancy however it should be used at the lowest effective dose for the shortest possible time and at the lowest possible frequency”). Taking these uncertainties into account, it is appropriate to include long-term outcome data into the already existing and ongoing studies and cohorts to generate data and create certainties on the existence and the extent of any potential negative effects. This can be done by integrating “pharmacovigilance” studies into long-term outcome studies, like renal outcome following neonatal ibuprofen exposure (81). The recent paper of Juurjärvi et al. on the post-discharge outcome (2 years) in a cohort of preterm neonates included in a prospective study on prophylactic paracetamol prescription to induce ductus arteriosus closure hereby serves as a relevant illustration that such data can be generated (82–84).

In conclusion, effective and safe pain management in neonates matters. This is not only related to ethical constraints or human empathy but even more because pain treatment is an important and crucial part of contemporary medical, paramedical, and nursing care to improve the outcome in neonatal intensive care graduates. Based on the available evidence, paracetamol has opioid-sparing effects for major pain syndromes, is effective to treat minor to moderate pain syndromes, but fails for procedural pain management in neonates. However, there are also upcoming association type of epidemiological studies on the relation between exposure to analgesics—including paracetamol—and the negative short- or long-term outcome characteristics (neuro-behavioral, atopy, and fertility). Because of these dual findings, further research is needed. This includes a search for other effective strategies to prevent or treat pain. This includes the collection of data on long-term outcome and also after paracetamol exposure to inform all relevant stakeholders on the efficacy/safety balance.

## Author Contributions

KA developed the concept, performed and interpreted the structured searches, and wrote and approved the paper.

### Conflict of Interest

The author declares that the research was conducted in the absence of any commercial or financial relationships that could be construed as a potential conflict of interest.
